# *TM6SF2* rs58542926 influences hepatic fibrosis progression in patients with non-alcoholic fatty liver disease

**DOI:** 10.1038/ncomms5309

**Published:** 2014-06-30

**Authors:** Yang-Lin Liu, Helen L. Reeves, Alastair D. Burt, Dina Tiniakos, Stuart McPherson, Julian B. S. Leathart, Michael E. D. Allison, Graeme J. Alexander, Anne-Christine Piguet, Rodolphe Anty, Peter Donaldson, Guruprasad P. Aithal, Sven Francque, Luc Van Gaal, Karine Clement, Vlad Ratziu, Jean-Francois Dufour, Christopher P. Day, Ann K. Daly, Quentin M. Anstee

**Affiliations:** 1 Institute of Cellular Medicine, The Medical School, Newcastle University, Newcastle upon Tyne NE2 4HH, UK; 2 Northern Institute for Cancer Research, The Medical School, Newcastle University, Newcastle upon Tyne NE2 4HH, UK; 3 Liver Unit, Department of Medicine, Addenbrooke’s Hospital, Cambridge CB2 0QQ, UK; 4 University Clinic of Visceral Surgery and Medicine, Inselspital Bern, 3010 Bern, Switzerland; 5 Institut National de la Santé et de la Recherche Médicale (INSERM), U1065, Team 8, Nice F-06204, Cedex 3, France; 6 NIHR Nottingham Digestive Diseases Biomedical Research Unit, Nottingham University Hospitals NHS Trust and University of Nottingham, Nottingham NG7 2UH, UK; 7 Department of Endocrinology, Diabetology and Metabolism, Antwerp University Hospital, Wilrijkstraat 10, B-2650 Edegem, Antwerp, Belgium; 8 Institute of Cardiometabolism and Nutrition, Pitié-Salpêtrière Hospital, 75013 Paris, France; 9 These authors jointly supervised this work; 10 Present address: School of Medicine, University of Adelaide, Eleanor Harrald Building, Frome Road, Adelaide, South Australia 5005, Australia

## Abstract

Non-alcoholic fatty liver disease (NAFLD) is an increasingly common condition, strongly associated with the metabolic syndrome, that can lead to progressive hepatic fibrosis, cirrhosis and hepatic failure. Subtle inter-patient genetic variation and environmental factors combine to determine variation in disease progression. A common non-synonymous polymorphism in *TM6SF2* (rs58542926 c.449 C>T, p.Glu167Lys) was recently associated with increased hepatic triglyceride content, but whether this variant promotes clinically relevant hepatic fibrosis is unknown. Here we confirm that *TM6SF2* minor allele carriage is associated with NAFLD and is causally related to a previously reported chromosome 19 GWAS signal that was ascribed to the gene *NCAN*. Furthermore, using two histologically characterized cohorts encompassing steatosis, steatohepatitis, fibrosis and cirrhosis (combined *n*=1,074), we demonstrate a new association, independent of potential confounding factors (age, BMI, type 2 diabetes mellitus and *PNPLA3* rs738409 genotype), with advanced hepatic fibrosis/cirrhosis. These findings establish new and important clinical relevance to *TM6SF2* in NAFLD.

## Abstract (web summary)

Non-alcoholic fatty liver disease (NAFLD) is characterized by increased hepatic triglyceride content (HTGC) in the absence of high alcohol consumption. Here the authors show that a genetic variant in *TM6SF2*, which is known to be associated with HTGC, is a clinically relevant modifier of hepatic fibrogenesis and increases the risk of progressive NAFLD.

## Graphical abstract

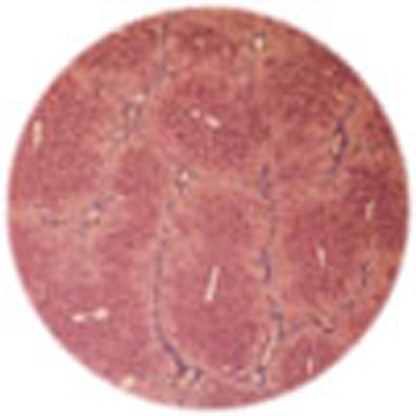

Non-alcoholic fatty liver disease (NAFLD) represents a spectrum of progressive liver disease characterized by increased hepatic triglyceride content (HTGC) in the absence of excess alcohol consumption^1^. NAFLD includes simple steatosis, non-alcoholic steatohepatitis, fibrosis and ultimately cirrhosis, and is strongly associated with features of the metabolic syndrome (obesity, insulin resistance/type 2 diabetes mellitus (T2DM) and dyslipidaemia)^1^. Reflecting the increasing prevalence of these conditions, NAFLD is estimated to affect approximately one-third of the population in many developed countries. Simple steatosis is generally considered to have a benign course and therefore to be of limited prognostic relevance^2^,^3^. However, some NAFLD patients exhibit progressive steatohepatitis leading to cirrhosis and/or hepatocellular carcinoma (HCC), conditions that confer increased morbidity and mortality^1^,^4^. Despite its high prevalence, only a minority of NAFLD patients progress to significant fibrosis and experience the associated morbidity^1^. Thus, similar to other common diseases (for example, obesity, T2DM and cardiovascular disease), NAFLD is best considered as a complex trait in which disease phenotype results from environmental exposures acting on a susceptible polygenic background that comprises multiple independent modifiers^5^,^6^,^7^.

Genome-wide association studies (GWAS)^8^,^9^,^10^ and candidate-gene studies^11^,^12^,^13^,^14^,^15^ have contributed greatly to our understanding of the genetic contribution to NAFLD pathogenesis and variability of prognosis (reviewed in ref. 7). Among the loci identified, the non-synonymous single-nucleotide polymorphism (SNP) in *PNPLA3* (rs738409 c.444 C>G, p.Ile148Met), *patatin-like phospholipase domain containing 3,* has been validated across multiple patient cohorts^8^,^9^,^12^,^16^. Importantly, carriage of this SNP has been robustly associated not only with steatosis but also with clinically relevant factors, including severity of hepatic fibrosis/cirrhosis and development of NAFLD-related HCC^12^,^17^,^18^.

Recently, Kozlitina *et al.*^19^ showed that a non-synonymous SNP in *TM6SF2* (rs58542926 c.449 C>T, p.Glu167Lys), *transmembrane 6 superfamily member 2*, a gene of unknown function on chromosome 19, was associated with proton magnetic resonance spectroscopy (^1^H-MRS) quantified HTGC based on genotyping with a genome-wide exome chip^19^. This variant has also been associated with dyslipidaemia and cardiovascular risk^20^. The *TM6SF2* rs58542926 SNP lies within 50 kb of an *NCAN* gene variant (rs2228603 c.274 C>T, p.Pro92Ser) that has previously been associated with HTGC in another GWAS^9^,^21^. Both SNPs are in strong linkage disequilibrium (D′=0.926, *r*^2^=0.798). Conditioning on the *TM6SF2* variant abrogated the effect of the *NCAN* variant while the reverse did not occur, suggesting that *TM6SF2* rs58542926 is more strongly associated with the HTGC phenotype. Homozygote *TM6SF2* rs58542926 minor (T) allele carriage was shown to be associated with a modest but statistically significant increase in ^1^H-MRS measured HTGC from 5.86±0.25% in CC homozygotes to 15.04±2.23% in TT homozygotes^19^. *In vitro* and *in vivo* functional studies also supported this conclusion but were unable to determine whether the effect of *TM6SF2* was limited to steatosis or had broader clinical relevance, as has already been shown for *PNPLA3* (ref. 19).

The aim of the current study was, first, to determine whether the association with NAFLD reported by Kozlitina *et al.*^19^ could be independently validated; and, second, to establish whether the *TM6SF2* rs58542926 variant was associated with clinically important disease end points that have prognostic relevance (in particular stage of hepatic fibrosis or development of NAFLD-related HCC). To address this, we perform a quantitative analysis within a well-characterized European Caucasian ‘discovery’ cohort with histologically characterized NAFLD, controlling for relevant co-morbidities and factors that have previously been linked with disease progression (age, gender, body mass index (BMI), presence of T2DM and *PNPLA3* rs738409 genotype), and replicate our findings in a separate histologically characterized European Caucasian ‘validation’ cohort. To discover whether the *TM6SF2* rs58542926 variant also confers an increased risk of NAFLD-related HCC, we perform a secondary case–control analysis comparing the overall ‘combined’ cohort of NAFLD patients to a cohort of NAFLD–HCC patients.

## Results

### Increased *TM6SF2* rs58542926 C>T minor allele carriage in NAFLD

In the NAFLD discovery cohort, the *TM6SF2* rs58542926 genotypes were confirmed to be in Hardy–Weinberg equilibrium with a minor allele frequency of 0.12, significantly higher than that observed in a reference Northern European population sample (MAF 0.07, http://browser.1000genomes.org) or a cohort of 265 Caucasian self-reported ‘healthy workers’ recruited from offices and factories locally in the North East of England (MAF 0.07) and so supportive of an association between these variants and NAFLD. Indeed, a gene-dosage effect was observed for both variants in the discovery cohort with the incidence of NAFLD increasing with the number of minor alleles possessed (*X*^2^ for trend, *P*=0.0008; Supplementary Table 1). A similar association was also confirmed for *PNPLA3* rs738409, *P*=0.0001 (Supplementary Table 2). Specific histological components of the NAFLD disease phenotype were next assessed individually.

### *TM6SF2* and degree of histological steatosis

As a positive control, and consistent with our previously reported analysis^12^, carriage of the *PNPLA3* rs738409 minor allele was significantly associated with degree of steatosis in multivariate analysis adopting an additive model adjusted for gender, age at biopsy, BMI and presence of T2DM (*β*=0.192±0.056, 95% confidence interval (CI) 0.082–0.301; *P*=6.74 × 10^−4^). However, in contrast to the report by Kozlitina *et al.*^19^, neither *TM6SF2* rs58542926 (*β*=0.087±0.083, 95%CI −0.076 to 0.250; *P*=0.296) nor *NCAN* rs2228603 (*β*=0.050±0.085, 95%CI −0.116 to 0.216; *P*=0.554) were found to be significantly associated with degree of histologically determined steatosis in the 349-patient discovery cohort. This was also the case in the 725-patient validation cohort (*P*=0.17). However, a trend towards significance was observed when the two cohorts were combined (*β*=0.111±0.059, 95%CI −0.0041 to 0.2268; *P*=0.053), suggesting that an underlying effect on degree of steatosis may be present but of relatively small size. An effect became apparent when the multivariate analysis in the combined cohort was repeated after subdividing the cohort into those with mild steatosis (S0–1) and pronounced steatosis (S2–3). Here, carriage of each copy of the *TM6SF2* rs58542926 C>T minor allele was associated with increased risk of greater steatosis (odds ratio (OR) 1.379, 95%CI 1.019–1.865; *P*=0.037), although with a marginal level of significance.

### *TM6SF2* and severity of histological steatohepatitis

Next, the association with steatohepatitis activity was tested using a composite score incorporating severity of necroinflammation and ballooning hepatocyte degeneration. *TM6SF2* rs58542926, but not *NCAN* rs2228603, was associated with severity of steatohepatitis in the discovery cohort by multivariate analysis adopting an additive model adjusted for gender, age at biopsy, BMI, T2DM and *PNPLA3* rs738409 genotype (*β*=0.288±0.139, 95%CI 0.015–0.561; *P*=0.039). However, this effect was not replicated in the validation or combined cohorts.

### *TM6SF2* and stage of histological fibrosis

Finally, the association with NAFLD fibrosis stage was tested. In the discovery cohort multivariate analyses adopting an additive model adjusted for gender, age at biopsy, BMI, T2DM and *PNPLA3* rs738409 genotype found that *TM6SF2* rs58542926 (*β*=0.549±0.135, 95%CI 0.285–0.813; *P*=5.57 × 10^−5^) and *NCAN* rs2228603 (*β*=0.419±0.138, 95%CI 0.148–0.689; *P*=0.0026) were both significantly associated with stage of fibrosis. The association between *TM6SF2* rs58542926 and fibrosis stage persisted when analysis included both the *NCAN* rs2228603 and the *PNPLA3* rs738409 SNPs as covariates (*β*=0.552±0.205, 95%CI 0.151–0.953; *P*=0.0074). However, the association with *NCAN* rs2228603 was lost when the analysis was conditioned on rs58542926. Thus, the association is driven by the *TM6SF2* rs58542926 variant, and carriage of its minor allele confers significantly greater NAFLD-related hepatic fibrosis independent of gender, age at biopsy, BMI, T2DM and *PNPLA3* rs738409 genotype.

This strong association between *TM6SF2* rs58542926 and fibrosis stage was replicated independently in the validation cohort (*β*=0.238±0.097, 95%CI 0.047–0.428; *P*=0.014) and also clearly demonstrated in the combined cohort (*β*=0.357±0.079, 95%CI 0.203–0.511; *P*=6.36 × 10^−6^) by using an additive model adjusted for gender, age at biopsy, BMI, T2DM and *PNPLA3* rs738409 genotype in both cases. To illustrate the potential clinical relevance of this finding, when the multivariate analysis was repeated subdividing the NAFLD cohort into those with mild fibrosis (F0–1) and advanced fibrosis (F2–4), carriage of each copy of the *TM6SF2* rs58542926 C>T minor allele was associated consistently with a significant increased risk of advanced fibrosis, independent of gender, age at biopsy, BMI, T2DM and *PNPLA3* rs738409 genotype across each cohort studied (Table 1).

### *TM6SF2* and risk of HCC

There is increasing evidence that NAFLD predisposes to an increased risk of HCC^22^, an effect influenced by *PNPLA3* rs738409 genotype independent of the presence of cirrhosis^18^. We therefore sought to determine whether *TM6SF2* rs58542926 had a similar effect. A cohort of 99 consecutive Northern European Caucasian patients with primary NAFLD-related HCC was identified according to the joint European Association for the Study of the Liver and European Association for the Research and Treatment of Cancer (EASL-EORTC) guidelines^23^. *TM6SF2* rs58542926 allele and genotype frequencies in this cohort were compared with the combined NAFLD cohort described above (*n*=1,074). In univariate analysis, homozygote carriage of the *TM6SF2* rs58542926 minor allele was associated with an increased risk of NAFLD–HCC with respect to CC (OR 1.922, 95%CI 1.31–2.81; *P*=6.81 × 10^−4^); however, significance was lost in multivariate analysis incorporating known risk factors including age, gender, BMI, T2DM and presence of cirrhosis (*P*=0.42).

## Discussion

The region on chromosome 19 (19p13) flanking *TM6SF2* has been reported to be associated with NAFLD^9^,^19^,^21^ as well as variations in plasma cholesterol, triglyceride and low-density lipoprotein levels^20^,^24^,^25^ in several previous studies. In particular, a variant within the *NCAN* gene (rs2228603 C>T) that is in strong linkage disequilibrium (D′=0.926, *r*^2^=0.798) with *TM6SF2* rs58542926 was reported to be associated with radiologically and histologically characterized NAFLD in both GWAS and candidate-gene studies^9^,^21^. Before the recent publication by Kozlitina *et al.*^19^, examination of linkage disequilibrium patterns across the region had already brought that association into question^7^. It was, however, the use of a genome-wide exome-chip genotyping approach, combined with detailed association analysis conditioning on previously published variants across the 19p13 region, that determined that the causative variant affecting HTGC was *TM6SF2* rs58542926 (ref. 19). When considered alongside a separate study by Holmen *et al.*^20^, which demonstrated an association with cardiovascular disease and circulating triglyceride/total cholesterol levels, it appears that *TM6SF2* rs58542926 C-allele carriage increases circulating triglyceride/total cholesterol while T-allele carriage promotes hepatic triglyceride/cholesterol retention. In clinical practice, simple steatosis is generally considered to have a benign course and so degree of HTGC is of limited prognostic relevance^1^,^3^,^26^. In contrast, progressive hepatic fibrosis leading to cirrhosis is the principal common pathway to hepatic failure and a liver-related death^2^,^3^. Using two large, well-characterized European Caucasian cohorts with biopsy-proven NAFLD, we demonstrate that carriage of the *TM6SF2* rs58542926 variant is strongly associated with the presence of NAFLD and, in particular, with a significantly greater risk of developing advanced hepatic fibrosis/cirrhosis.

Evidence to support a modifier effect of the *TM6SF2* rs58542926 variant on histologically determined HTGC (steatosis), seen only when the 1,074-patient strong combined cohort was studied, is arguably more modest than might be expected. Our findings do support the previously reported association^9^,^19^,^21^, although differences in sensitivity to subtle changes in HTGC between radiological and histological modalities may have reduced the power to detect this effect^27^. Kozlitina *et al.*^19^ reported that the maximal effect of the *TM6SF2* variant in European Caucasians was only a mean 9.2% increase in ^1^H-MRS quantified HTGC in TT homozygotes above the ~5.9% observed in CC homozygotes^19^. Histological assessment of hepatic steatosis uses broad microscopic categories reflecting the proportion of hepatocytes that are visibly steatotic (S0 <5%, S1 5–33%, S2 33–66% and S3 >66%)^28^. Based on data from previous comparative modality analysis^27^, the modest gene effect size reported by Kozlitina *et al.*^19^ (less than a threefold increase in HTGC above normal) would likely be encompassed within the histological S1 bracket, and therefore may not be apparent histologically. Combined with the relatively low minor allele frequency in the background population, smaller cohorts may therefore have insufficient statistical power for an association to become evident.

The modifier effect of the *TM6SF2* variant on grade of steatohepatitis (disease activity) was apparent in the initial discovery cohort analysis; however, statistical significance was not reached in the subsequent validation analysis. The validation cohort comprised a mixture of patients recruited from both hepatology and bariatric services and, although the cohorts appear well matched histologically, the validation cohort exhibited higher mean BMI levels (38.5±9.1 versus 34.5±5.7 kg m^−2^, *P*<0.0001), a younger mean age (47.6±12.4 versus 51.5±12.0 years, *P*<0.0001), a greater female preponderance (56.1 versus 32.1%, *P*<0.0001) and a lower prevalence of T2DM (32.4 versus 46.1%, *P*<0.0001) than the discovery cohort (Table 2). These factors may have impacted on our ability to replicate the initial association with steatohepatitis in a multivariate analysis. Further study of the variant in other patient cohorts and exploration of the functional effects of *TM6SF2* on inflammatory response will be needed to address this point.

The key finding of the current study is that carriage of the *TM6SF2* rs58542926 C>T minor allele is unequivocally associated with an increased risk of advanced NAFLD-associated hepatic fibrosis. This highly significant effect was consistently demonstrated across all the cohorts studied and was independent of potentially confounding factors including gender, age at time of biopsy, BMI, T2DM and *PNPLA3* rs738409 genotype. Conditional analysis undertaken as part of the present study adds further weight to the assertion that the 19p13 signal is causally related to *TM6SF2* and not *NCAN*, not only for HTGC as was previously reported^19^ but now also for stage of hepatic fibrosis. These findings therefore establish a new and important clinical relevance to the recently described association between *TM6SF2* and NAFLD, and suggest that *TM6SF2* should be considered alongside *PNPLA3* (refs 8, 12) and *GCKR*^9^,^29^,^30^, as one of a handful of genes so far identified that are associated not only with variations in hepatic triglyceride accumulation but also with fibrogenesis^7^. It is noteworthy that across all the cohorts studied, the OR for advanced fibrosis conferred by each copy of the *TM6SF2* variant carried was consistently of similar or up to twofold greater magnitude than that which was observed, or has previously been reported^12^,^31^, for the widely studied *PNPLA3* rs738409 variant (Table 1).

Carriage of the *TM6SF2* variant was associated with increased risk of progression to NAFLD–HCC in univariate analysis. In contrast to *PNPLA3* (ref. 18), this effect was not sustained when confounding factors including age, T2DM and presence of underlying cirrhosis were included in the model. Carriage of the *TM6SF2* variant therefore does not appear to further increase HCC risk independent of its effect on fibrosis stage. It should, however, be noted that the NAFLD–HCC cohort contained only 99 patients and so, combined with a relatively modest TM6SF2 rs58542926 minor allele frequency, the current study had ~70% power to detect an effect if an additive genetic model and risk similar to that seen for fibrosis is assumed (*α*=0.05)^32^. An association cannot therefore be completely excluded, but would seem unlikely. Studies using larger cohorts of NAFLD–HCC patients than are presently available will be required to provide sufficient power to study this further.

The *TM6SF2* rs58542926 c.449 C>T variant is a non-synonymous change producing a glutamate to lysine amino-acid substitution at residue 167 (Glu167Lys), which is highly conserved across mammals^19^. First identified as part of a large-scale sequencing project, little is currently known about the biological function of the TM6SF2 protein product^33^. Adenovirus-mediated short hairpin RNA knockdown of *Tm6sf2* in mice has been shown to increase HTGC and reduce very low-density lipoprotein (VLDL) secretion, suggesting that *TM6SF2* activity is necessary for normal VLDL secretion, and that impaired *TM6SF2* function causally contributes to NAFLD^19^. However, these *in vivo* studies were of too short a duration to adequately address the effects on steatohepatitis or fibrogenesis. Furthermore, previous experimental evidence has shown that hepatic triglyceride accumulation may not itself be directly hepatotoxic. This was elegantly demonstrated in mice by silencing hepatic gene expression of *diacylglycerol O-acyltransferase 2* (*Dgat2*), a key enzyme mediating the conversion of free fatty acids to triglyceride^34^. Rather than ameliorating steatohepatitis, the consequent reduction in hepatocyte triglyceride synthesis was associated with increased fatty acid oxidation, particularly through Cyp2e1, leading to greater oxidative stress, cellular damage and higher serum transaminase levels^34^. It is therefore tempting to speculate that the function of *TM6SF2* and the mechanism through which *TM6SF2* drives NAFLD-associated hepatic fibrosis may be other than through increased triglyceride accumulation.

In conclusion, the current study confirms that *TM6SF2* is associated with histologically defined NAFLD, and is the first demonstration that this gene serves as a powerful modifier of hepatic fibrogenesis. That this gene is also associated with disturbed cholesterol metabolism and so may modify risk of cardiovascular events including myocardial infarction^20^ suggests that *TM6SF2* is an important determinant of clinical outcome across several facets of metabolic syndrome-related end-organ damage. In light of evidence that cholesterol accumulation in hepatic stellate cells promotes NAFLD fibrosis^35^, it is tempting to speculate that TM6SF2 may act as a ‘switch’ with *TM6SF2* rs58542926 T-allele-mediated hepatic retention of triglyceride and cholesterol predisposing to NAFLD fibrosis while C-allele carriage promotes VLDL excretion, protecting the liver at the expense of increased risk of cardiovascular disease. These data mandate further mechanistic study to determine the physiological and pathophysiological role of this gene in various tissues and cell types as a modifier of fibrogenesis and a putative therapeutic target.

## Methods

### Patients

Patients were recruited from hepatology clinics at several European specialist centres: the Freeman Hospital, Newcastle upon Tyne, UK; Addenbrooke’s Hospital, Cambridge, UK; Nottingham University Hospitals NHS Trust, Nottingham, UK; Inselspital Hospital, Bern, Switzerland; Antwerp University Hospital, Belgium; and Pitié-Salpêtrière Hospital, Paris, France. The study had all the necessary ethical approvals (UK: Newcastle and North Tyneside 1 REC (10/H0906/41), Norfolk REC (06/Q0106/70)] and Nottingham 2 REC (GM010201); Switzerland: Inselspital Bern Local Ethics Committee; Belgium: Antwerp University Hospital Ethics Committee; France: CPP (Comité de Protection des Personnes) Paris VI IDF Pitié—Salpêtrière Hospital). All participants gave informed consent. In all cases, alternative diagnoses were excluded, including excess alcohol intake (alcohol intake <20 g per day for women; and <30 g per day for men), chronic viral hepatitis (hepatitis B and hepatitis C), autoimmune liver diseases, hereditary hemochromatosis, α1-antitrypsin deficiency, Wilson’s disease and drug-induced liver injury. Clinical and laboratory data were collected at the time of diagnosis including basic anthropometrics so that BMI could be calculated, and relevant co-morbidity including the presence of T2DM (fasting glucose ≥7.1 mmol l^−1^ (≥128 mg dl^−1^) or treatment with anti-diabetic drugs) and evidence of underlying cirrhosis was recorded. The degree of steatosis (S0–3), activity of steatohepatitis (A0–4) and stage of fibrosis (F0–4) were scored according to the validated semi-quantitative SAF score^28^. The main study cohorts were:

- An initial discovery cohort of 349 consecutive European Caucasian patients from the United Kingdom with histologically characterized NAFLD of different stages of disease. These were unrelated patients with histologically characterized NAFLD, derived from a patient population originally identified as having ultrasonographically detected bright liver and abnormal biochemical tests (alanine transaminase and/or gamma-glutamyl transferase).
- A validation cohort of 725 consecutive European Caucasian patients from centres in UK and mainland Europe with histologically characterized NAFLD of different stages of disease. Patients in this cohort were unrelated patients with histologically characterized NAFLD, derived from a patient population originally identified as having ultrasonographically detected bright liver and abnormal biochemical tests (alanine transaminase and/or gamma-glutamyl transferase) or identified as having evidence of NAFLD at the time of bariatric surgery.

Together, these comprised the combined cohort of 1,074 patients with histologically characterized NAFLD. Demographic and histological details are shown in Table 2. A description of the ‘healthy workers’ cohort recruited in the North East of the United Kingdom has previously been published^36^.

A separate cohort of 99 consecutive Northern European Caucasian patients with primary HCC arising on a background of NAFLD was identified (NAFLD–HCC cohort). The diagnosis of HCC was established histologically or through non-invasive assessment according to the EASL–EORTC clinical practice guidelines^23^. The presence of NAFLD was determined through histological assessment of non-tumour liver tissue or, when biopsy was not clinically appropriate, through radiological evidence of hepatic steatosis.

### Liver biopsy

Liver biopsy was performed under radiological guidance. Specimens (at least 1.6 cm length and 1.5 mm thick) were fixed in 10% neutral formalin for evaluation and embedded in paraffin for histological examination. Tissue sections were stained with haematoxylin and eosin, impregnated with silver for visualizing reticulin framework and stained with Sirius Red Fast Green for visualizing collagen. Liver biopsies were reviewed by a single expert liver pathologist at each participating centre, unaware of clinical or genetic data. The degree of steatosis (S0–3), activity of steatohepatitis (A0–4) and stage of fibrosis (F0–4) were scored according to the validated semi-quantitative SAF score (Supplementary Table 3)^28^,^37^. In 25 HCC patients, the diagnosis of HCC was confirmed histologically and graded according to Edmondson and Steiner^38^, adapted for needle biopsy specimens.

### DNA preparation

Venous blood was collected from each patient and DNA was prepared from peripheral blood lymphocytes using a perchlorate–chloroform isolation method^39^. In brief, 35 ml lysis buffer (10 mM Tris–HCl (pH 8.0), 320 mM sucrose, 5 mM magnesium chloride and 1% Triton X-100) was added to 5 ml venous blood in a 50-ml polypropylene centrifuge tube. After mixing, the tube was centrifuged at 3,000 *g* for 10 min. The supernatant was discarded and the cell pellet was re-suspended in 2 ml of solution B (400 mM Tris–HCl (pH 8.0) 6 0 mM EDTA, 150 mM NaCl and 1% SDS). A quantity of 500 μl of sodium perchlorate (5 M) was added and the sample was mixed at room temperature for 15 min before incubating in a preheated hot block at 65 °C for 30 min. Next, 2 ml of chloroform was added and the sample was mixed for 10 min at room temperature. The tube was then centrifuged at 1,400*g* for 10 min, and the upper, clear DNA-containing phase was transferred to a new 15 ml polypropylene tube. Two volumes of cold ethanol were added to the aqueous phase, and the tube was gently inverted until the DNA precipitated. The DNA was spooled using a soft plastic sterile loop and allowed to air dry for 20 min. DNA was then re-suspended by incubation in 200 μl water at 60 °C. Samples are quantitated and quality assessed by absorbance measurements at 260 and 280 nm. Genotyping was performed by personnel unaware of clinical status or histology of patients.

### *TM6SF2* rs58542926, *NCAN* rs2228603 and *PNPLA3* rs738409 genotyping

*TM6SF2* rs58542926, *NCAN* rs2228603 and *PNPLA3* rs738409 genotypes were determined by allelic discrimination using TaqMan reagents (Applied Biosystems Inc., USA) according to the manufacturer’s protocol. Control samples of known genotype were also included in every 96-well plate (blank, homozygous wild-type, homozygous mutant and heterozygous).

### Statistical analysis

Statistical analyses were performed using SPSS v19.0 (IBM, USA) to collate and analyse cohort phenotype data. Continuous variables were tested using Student’s *t*-test/one-way analysis of variance and categorical variables by *χ*^2^-squared test unless otherwise stated. PLINK v1.07 (ref. 40) (via the gPLINK v2.050 GUI) was used to conduct the genetic analysis. An initial univariate *χ*^2^-squared analysis was performed. Subsequently, multivariate logistic regression analysis was conducted incorporating biologically relevant covariates that were associated with risk of NAFLD progression (age, gender, BMI, presence of T2DM and *PNPLA3* rs738409 genotype) to test the genetic association. An additive genetic model best fitted the data and was reported. Results are expressed as beta *β*±s.e.m. or OR with 95%CI as appropriate. Significance was taken as *P*<0.05 throughout.

## Author contributions

Q.M.A., A.K.D. and C.P.D. conceived the research. Clinical phenotype data collation and sample acquisition/DNA preparation was performed by J.B.S.L., Q.M.A., C.P.D., S.M., H.L.R., M.E.D.A., G.J.A., A.-C.P., P.D., G.P.A., S.F., L.V.G., K.C., V.R. and J.-F.D. Genotyping was performed by Y.-L.L., and assisted by J.B.S.L. and RA. Histological analysis of tissues and scoring was conducted by A.D.B. and D.T. Statistical analysis and interpretation of results was performed by Y.-L.L., A.K.D. and Q.M.A. The manuscript was written and revised by Q.M.A., A.K.D. and Y.-L.L. All authors critically reviewed the manuscript for important intellectual content and approved the final submitted manuscript.

## Additional information

**How to cite this article:** Liu, Y.-L. *et al.* *TM6SF2* rs58542926 influences hepatic fibrosis progression in patients with non-alcoholic fatty liver disease. *Nat. Commun.* 5:4309 doi: 10.1038/ncomms5309 (2014).

## Supplementary Material

Supplementary Information Supplementary Tables 1-3 and Supplementary References

## Figures and Tables

**Table 1:** Multivariate analysis of association between *TM6SF2* rs58542926 genotype and fibrosis stage F0–1 (mild) versus F2–4 (advanced).

**Variables**	**Discovery cohort (*****n*****=349)**		**Validation cohort (*****n*****=725)**		**Combined cohort (*****n*****=1,074)**	
	**OR (95%CI)**	***P*****-value**	**OR (95%CI)**	***P*-value**	**OR (95%CI)**	***P*****-value**
*TM6SF2* genotype	2.94 (1.76–4.89)	3.44 × 10^−5^	1.46 (1.03–2.09)	0.0362	1.88 (1.41–2.5)	1.63 × 10^−5^
*PNPLA3* genotype	1.57 (1.21–2.19)	0.0086	1.32 (1.05–1.66)	0.0183	1.40 (1.16–1.69)	4.84 × 10^−4^
Age	1.03 (1.01–1.06)	0.0045	1.02 (1.01–1.04)	0.0041	1.03 (1.01–1.04)	1.57 × 10^−5^
Gender (female)	0.94 (0.57–1.56)	0.8297	1.81 (1.30–2.50)	4.50 × 10^−4^	1.43 (1.09–1.89)	0.0096
BMI	1.05 (1.00–1.10)	0.0368	1.03 (1.01–1.05)	9.80 × 10^−4^	1.04 (1.02–1.05)	3.78 × 10^−5^
T2DM	2.39 (1.49–3.84)	0.0003	2.73 (1.93–3.88)	1.68 × 10^−8^	2.57 (1.95–3.39)	1.78 × 10^−11^

BMI, body mass index; CI, confidence interval; OR, odds ratio; T2DM, type 2 diabetes mellitus.

Additive model including age, gender, BMI, T2DM and *PNPLA3* rs738409 genotype as covariates. Discovery/validation/combined cohorts: stage F0–1 (mild) *n*=198/439/637, stage F2–4 (advanced) *n*=151/286/437.

**Table 2:** Demographic characteristics of patient cohorts.

	**Discovery cohort**	**Validation cohort**	**Combined cohort**	**NAFLD–HCC cohort**
Number	349	725	1,074	99
Ethnicity	European Caucasian	European Caucasian	European Caucasian	European Caucasian
Gender (female)	147 (42.1%)	407 (56.1%)	554 (51.6%)	19 (19.2%)
Age, years	51.5±12.0	47.6±12.4	48.9±12.4	70.5±8.0
BMI, kg m^−2^	34.5±5.7	38.5±9.1	37.2±8.3	31.9±6.7
T2DM (yes)	161 (46.1%)	235 (32.4%)	396 (36.9%)	68 (68.7%)
Steatosis score*				
S0	5 (1.4%)	60 (8.3%)	65 (6.1%)	—
S1	99 (28.4%)	206 (28.4%)	305 (28.4%)	—
S2	166 (47.6%)	247 (34.1%)	413 (38.5%)	—
S3	79 (22.6%)	204 (28.1%)	283 (26.4%)	—
Activity score (composite hepatocyte ballooning and necroinflammation scores)*				
A0	81 (23.2%)	132 (18.2%)	213 (19.8%)	—
A1	65 (18.6%)	133 (18.3%)	198 (18.4%)	—
A2	101 (28.9%)	214 (29.5%)	315 (29.3%)	—
A3	64 (18.3%)	149 (20.6%)	213 (19.8%)	—
A4	31 (8.9%)	89 (12.3%)	120 (11.2%)	—
*Fibrosis score*				
F0	108 (30.9%)	277 (38.2%)	385 (35.8%)	
F1	90 (25.8%)	162 (22.3%)	252 (23.5%)	Non-cirrhotic: 32 (32.3%)
F2	55 (15.8%)	161 (22.2%)	216 (20.1%)	
F3	66 (18.9%)	75 (10.3%)	141 (13.1%)	
F4 (cirrhosis)	30 (8.6%)	50 (6.9%)	80 (7.4%)	Cirrhotic: 67 (67.7%)

BMI, body mass index; HCC, hepatocellular carcinoma; NAFLD, non-alcoholic fatty liver disease; T2DM, type 2 diabetes mellitus.

^*^Steatosis and activity score data incomplete in 8 (0.7%) and 15 (1.3%) of samples, respectively.
