# Supplementary material for: TM6SF2 rs58542926 influences hepatic fibrosis progression in patients with non-alcoholic fatty liver disease
Source: Nat Commun. 2014 Jun 30;5:4309. doi: 10.1038/ncomms5309 (PMC4279183; doi:10.1038/ncomms5309)
Supplement: Supplementary Information — Supplementary Tables 1-3 and Supplementary References [file ncomms5309-s1.pdf]

SUPPLEMENTARY INFORMATION

Supplementary Table 1: Genotype frequency of *TM6SF2* rs58542926 in Discovery Cohort NAFLD patients vs. the 1000 Genomes European Caucasian population sample.

TM6SF2 Genotypes	NAFLD N=349 (%)	EUR Pop. N=379 (%)	OR (95% CI)	P-value
**CC**	271 (77.6%)	328 (86.5%)	-	-
**CT**	70 (20%)	49 (12.9%)	OR 1.72 (1.16-2.57)	**0.008**
**TT**	8 (2.3%)	2 (0.5%)	OR 4.84 (1.01-22.9)	**0.049**

ChiSq for trend p=0.0008 (X^2=11.34, df=1).
1000 Genomes MAF (EUR) = 0.07 (http://browser.1000genomes.org).

Supplementary Table 2: Genotype frequency of *PNPLA3* rs738409 in Discovery Cohort NAFLD patients vs. the 1000 Genomes European Caucasian population sample.

PNPLA3 Genotypes	NAFLD N=349 (%)	EUR Pop. N=379 (%)	OR (95% CI)	P-value
**CC**	152 (43.5%)	233 (61.5%)	-	-
**CG**	148 (42.4%)	128 (33.8%)	OR 1.77 (1.29-2.42)	**0.0003**
**GG**	49 (14%)	18 (4.7%)	OR 4.17 (2.34-7.43)	**3.04×10^{-7}**

ChiSq for trend p<0.0001 (X^2=31.29, df=1).
1000 Genomes MAF (EUR) = 0.22 (http://browser.1000genomes.org).

Supplementary Table 3: Comparison of the FLIP SAF Score and the NAFLD CRN (Kleiner) Score for the histological grading and staging of NAFLD/NASH.

SAF Score[1, 2]			Kleiner Score[3]		
**Histological Feature**	**Category Definition**		**Histological Feature**	**Category Definition**	
**Steatosis**	0	<5%	**Steatosis**	0	<5%
	1	5-33%		1	5-33%
	2	34-66%		2	34-66%
	3	>66%		3	>66%
**(S) Steatosis Score 0-3**			***PLUS***		
**Hepatocyte Ballooning**	0	None	**Hepatocyte Ballooning**	0	None
	1	Clusters of hepatocytes with rounded shape and pale cytoplasm		1	Few
	2	Same as grade 1 with enlarged hepatocytes (>2x normal size)		2	Many
PLUS			***PLUS***		
**Inflammation**	0	None	**Inflammation**	0	None
	1	< 2 foci per 20x field		1	1–2 foci per x20 field
	2	> 2 foci per 20x field		2	2–4 foci per x20 field
				3	>4 foci per x20 field
**(A) Total = Activity Score 0-4**			**(NAS) Total = NAFLD Activity Score 0-8**		
**Fibrosis**	0	No fibrosis	**Fibrosis**	0	No fibrosis
	1a	Zone 3 mild perisinusoidal fibrosis		1a	Zone 3 mild perisinusoidal fibrosis
	1b	Zone 3 moderate perisinusoidal fibrosis		1b	Zone 3 moderate perisinusoidal fibrosis
	1c	Periportal/portal fibrosis only		1c	Periportal/portal fibrosis only
	2	Zone 3 plus portal/periportal fibrosis		2	Zone 3 plus portal/periportal fibrosis
	3	Bridging fibrosis		3	Bridging fibrosis
	4	Cirrhosis		4	Cirrhosis
**(F) Fibrosis Stage 0-4**			**Fibrosis Stage 0-4**		

Table modified from (Dyson et al, 2013[4]) with permission.

SUPPLEMENTARY REFERENCES

1. Bedossa P*, et al.* Histopathological algorithm and scoring system for evaluation of liver lesions in morbidly obese patients. *Hepatology* **56**, 1751-1759 (2012).
2. Bedossa P*, et al.* Utility and appropriateness of the FLIP algorithm and SAF score in the evaluation of biopsies of nonalcoholic fatty liver disease. *Hepatology*, (2014).
3. Kleiner DE*, et al.* Design and validation of a histological scoring system for nonalcoholic fatty liver disease. *Hepatology* **41**, 1313-1321 (2005).
4. Dyson JK, McPherson S, Anstee QM. Non-alcoholic fatty liver disease: non-invasive investigation and risk stratification. *Journal of clinical pathology* **66**, 1033-1045 (2013).
